# 3D Virtual reality assessment of right ventricle-pulmonary artery conduit dilation and coronary compression risk: a retrospective bi-center feasibility study

**DOI:** 10.3389/fcvm.2026.1768043

**Published:** 2026-04-10

**Authors:** David Hochstein, Yona Rothé, Avshalom Shaffer, Yisrael Parmet, Oliana Vazhgovsky, Hanita Shai, Alona Raucher, Racheli Sion Sarid, Erica Pollak, Dor Freidin, Lior Sasson, Yishai Salem, Uriel Katz, Sagi Asa, Netanel Nagar, Shai Tejman-Yarden, Sharon Borik Chiger

**Affiliations:** 1 Internal Medicine Department C, Sheba Medical Center, Ramat Gan, Israel; 2 Engineering in Medicine Lab (EiM), Sheba Medical Center, Ramat Gan, Israel; 3 The School of Medicine, Tel Aviv University, Tel Aviv, Israel; 4 Internal Medicine Department A, Shamir (Assaf Harofeh) Medical Center, Zerifin, Israel; 5 Department of Industrial Engineering and Management, Ben Gurion University, Beer Sheva, Israel; 6 Pediatric Cardiology, The Sylvan Adams Children’s Hospital, Wolfson Medical Center, Holon, Israel; 7 Pediatric Intensive Care Unit, The Sylvan Adams Children’s Hospital, Wolfson Medical Center, Holon, Israel; 8 Department of Radiology, Wolfson Medical Center, Holon, Israel; 9 Department of Plastic and Reconstructive Surgery, Sheba Medical CenterRamat Gan, Israel; 10 Cardiothoracic Surgery, Wolfson Medical Center, Holon, Israel; 11 International Congenital Heart Center, The Edmond and Lily Safra Children’s Hospital, Sheba Medical Center, Ramat Gan, Israel

**Keywords:** 3D reconstruction, congenital heart defect (CHD), pre-procedural planning, pulmonary artery conduit, virtual reality

## Abstract

**Background:**

Right ventricle-to-pulmonary artery (RV–PA) conduits are crucial to establishing or restoring RV–PA continuity in children with complex congenital heart disease. Progressive conduit obstruction is common, particularly in growing patients, and may necessitate transcatheter dilation and stenting. One of the major procedural concerns in these cases is the risk of coronary artery compression during stent implantation. This study evaluated the technical feasibility and clinical utility of patient-specific three-dimensional (3D) reconstruction and virtual reality (VR) modeling to enhance pre-procedural planning and coronary risk assessment.

**Methods:**

This retrospective bi-center feasibility analysis of pediatric patients who underwent evaluation for RV-PA conduit dilation and stenting was conducted at the Sheba and Wolfson Medical Centers, Israel, between January 2018 and September 2022. For 19 eligible patients, cardiac CT datasets were processed to generate high-fidelity 3D VR models. Two independent cardiologists assessed the models, quantified the distances between the conduit and the major coronary arteries before and after simulated balloon expansion, and provided structured qualitative feedback on VR usability.

**Results:**

VR-based anatomical measurements demonstrated strong inter-operator agreement (intraclass correlation coefficient >0.7 across most parameters). Both cardiologists rated VR significantly superior to CT alone for delineating coronary trajectories and assessing compression risk (mean score 4.58 vs. 3.78, *p* < 0.0001). VR model generation was technically successful in all cases, with intuitive user interface performance and rapid rendering times.

**Conclusions:**

Patient-specific 3D VR modeling is technically feasible and provides clinically meaningful advantages for planning RV-PA conduit interventions. VR enhances visualization of complex coronary anatomy beyond what is achievable with standard CT imaging and may support more accurate risk stratification, improved procedural planning, and potentially reduce catheterization-associated complications. These preliminary findings support further prospective evaluation for the integration of VR tools into routine congenital cardiac practice.

## Introduction

Right ventricle-to-pulmonary artery (RV-PA) conduits are widely used across all age groups as a surgical strategy to restore pulmonary blood flow in congenital heart diseases (CHDs) characterized by right ventricular outflow tract (RVOT) obstruction. These include pulmonary valve atresia, Tetralogy of Fallot (TOF), common arterial trunk, transposition of the great arteries with ventricular septal defect (VSD), and others (1–7). The procedure is feasible from the neonatal period and is associated with five-year survival rates exceeding 80% (1, 6).

Despite their clinical utility, RV-PA conduits require ongoing maintenance and periodic dilation and replacement, in that progressive stenosis, degeneration, calcification, or somatic outgrowth commonly develop during childhood, and have adverse hemodynamic consequences. Reported conduit durability varies widely, with 55%–95% remaining functional at five years, depending on patient- and conduit-specific factors (8–11). Transcatheter balloon dilation and stent implantation have therefore become essential modalities to prolonging conduit lifespan with reports indicating a median stent patency of approximately 6.9 years (12). However, conduit stenting carries the potentially catastrophic risk of compression of adjacent coronary arteries, particularly when the conduit lies in close proximity to the RVOT or ascending aorta (12, 13). As a result, selective coronary and aortic root angiography during balloon inflation is routinely performed to assess the coronary compression risk before stent deployment.

Advances in three-dimensional (3D) reconstruction and virtual reality (VR) technologies have begun to transform cardiovascular procedural planning (14–16). There has been growing interest in the utility of VR in medical training, pre-procedural guidance, immersive anatomical visualization, and patient education (17). In interventional cardiology, where procedural success often depends on accurately interpreting complex 3D anatomy from multimodal imaging, VR has substantial advantages. Recent studies have shown that VR can enhance planning for transcatheter aortic valve replacement (TAVR) by improving understanding of procedural steps and device positioning, and can support percutaneous coronary interventions in patients with anomalous coronary origins (18–20). VR also contributes to reducing radiation exposure by enabling anatomical angle assessment without the need for full rotational fluoroscopy (21).

**Figure 1:**
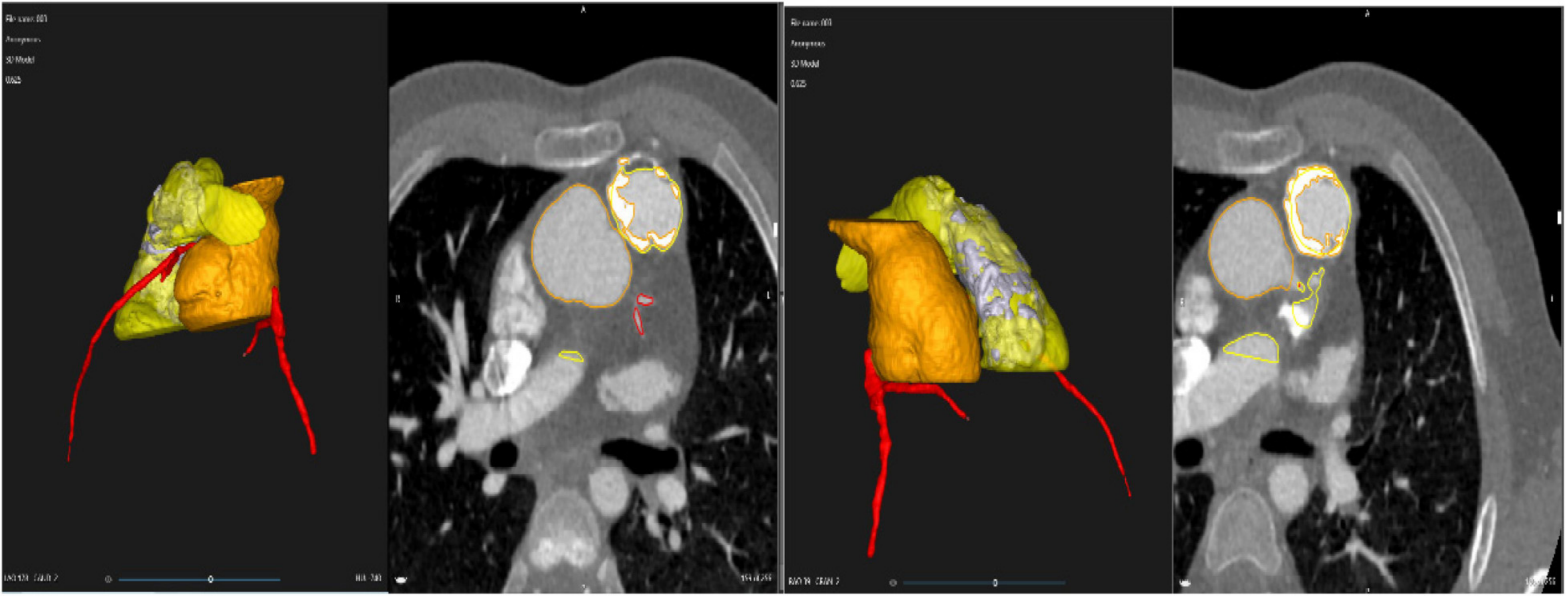
2D segmentation and 3D representation from a 12-year-old patient with a calcified homograft following repair of tetralogy of fallot. The patient subsequently underwent re-stenting for conduit stenosis and Melody valve implantation. Labels: The ascending aorta (Orange) the RV-PA conduit (yellow) and the coronary arteries (red).

**Figure 2:**
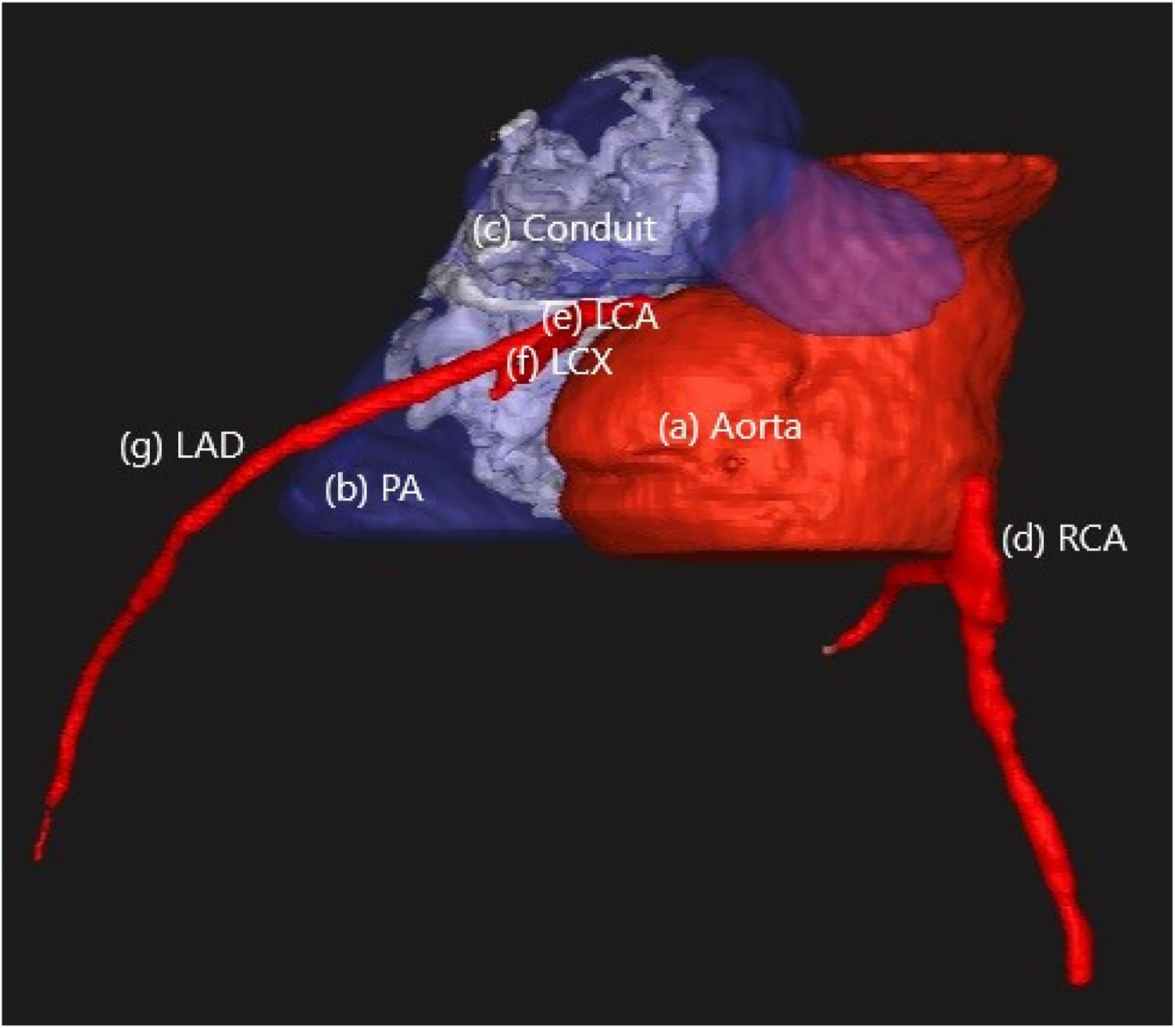
Anatomical annotations of 3D rendered segmentation; **(a)** aorta; **(b)** pulmonary artery (PA); **(c)** right ventricle–pulmonary artery conduit (RV–PA); **(d)** right coronary artery (RCA); **(e)** left coronary artery (LCA); **(f)** left circumflex artery (LCx); **(g)** left anterior descending artery (LAD).

Modern VR software makes it possible to convert conventional CT or MRI datasets into high-fidelity 3D models that can be manipulated in real time. These patient-specific reconstructions permit a detailed visualization of the RV-PA conduit, the coronary arteries, the ascending aorta and surrounding structures, thus providing an intuitive and immersive tool for pre-procedural assessment (Figures 1, 2). VR-based exploration is thus likely to be particularly advantageous in evaluating the spatial relationships that determine coronary compression risk during RV-PA conduit dilation and stenting, both of which can be challenging to interpret on standard cross-sectional imaging alone.

In this bi-center feasibility study, we assessed whether patient-specific VR reconstructions could reliably characterize the anatomical relationships between RV-PA conduits and adjacent coronary arteries, and whether this technology could enhance clinical decision-making for RV-PA conduit stenting. Specifically, we investigated the feasibility, inter-operator reliability, and potential clinical value of integrating VR into pre-procedural planning workflows for this high-risk population.

## Methods

This retrospective bi-center feasibility study was approved by the institutional review boards of Sheba Medical Center and Wolfson Medical Center. The cohort included 19 consecutive pediatric patients who underwent evaluation for RV-PA conduit dilation between January 2018 and September 2022 (13 from Sheba Medical Center and 6 from Wolfson Medical Center). All patients had clinically indicated cardiac CT scans performed.

The primary feasibility endpoints were:

1. Technical feasibility—successful generation of patient-specific 3D models from routine clinical CT datasets.
2. Inter-operator reliability—reproducibility of anatomical measurements by two independent cardiologists.
3. System usability—operator-reported ease of use and workflow integration.
4. Clinical utility—perceived added value of VR visualization compared with conventional CT imaging for procedural planning.

### CT image acquisition and 3D modeling

Computed tomography imaging was performed using 16-channel or higher multidetector CT scanners (Brilliance iCT; Philips Healthcare, Cleveland, OH, USA) with standardized acquisition parameters of 120 kVp and 100 mAs. All scans were obtained during systole 40%–50% of the cardiac cycle and in the supine position and interpreted by board-certified radiologists using Carestream Vue PACS. For the purposes of this study, only axial source images were used for subsequent anatomical analysis and 3D reconstruction. All CT scans were of high enough quality for 3D reconstruction and modeling.

The CT datasets were exported in DICOM format and processed using dedicated 3D reconstruction software to generate patient-specific virtual models of the RV-PA conduit, adjacent coronary arteries, and surrounding cardiovascular structures. These models served as the basis for virtual reality (VR) visualization and simulation analyses performed in the subsequent stages of the study. The duration of dedicated 3D reconstruction per patient estimated to approximately 60 min whereas the subsequent simulation analyses took 15 min per patient.

The VR simulated models were created from CT scan DICOM data using D2P® software (3D Systems Inc., Littleton, CO, USA) for segmentation. The resulting mesh files were converted to stereolithography (.STL) format files depicting 3D surface geometry. This enabled stereoscopic examination using a VR headset (Vive System, HTC, San Francisco, CA, USA) (Figure 1).

### RV-PA conduit analysis

For each patient, a detailed cardiovascular anatomy was reconstructed, including the RV-PA conduit, main and branch pulmonary arteries, ascending aorta, and the major coronary arteries; i.e., the left main coronary artery (LCA), left anterior descending artery (LAD), left circumflex artery (LCX), and right coronary artery (RCA) (Figure 2). The segmented DICOM datasets were processed to generate high-fidelity three-dimensional models that preserved vessel geometry and spatial relationships relevant to procedural planning.

The VR software enabled a dynamic graphical simulation of balloon dilation within the reconstructed RV-PA conduit. Balloon expansion was incrementally modeled in 1 mm diameter steps, generating real-time 3D representations of conduit deformation across the inflation range typically used in clinical stenting procedures. This functionality allowed the operators to visualize changes in conduit geometry, assess proximity between the expanding conduit and adjacent coronary arteries, and estimate the threshold at which clinically significant compression might occur.

For each case, the simulation supported:

1. A quantitative assessment of the minimum distances between the conduit and individual coronary arteries before and during simulated expansion.
2. A qualitative evaluation of coronary compression risk based on anatomical deformation patterns.
3. An estimation of the maximum safe balloon inflation diameter before potential coronary compromise.

This VR-based workflow enabled a comprehensive pre-procedural assessment of the conduit–coronary coronary artery proximity, compression risk evaluation, and maximum safe inflation estimation for each case.

### Measurement protocol and feasibility assessment

**Figure 3:**
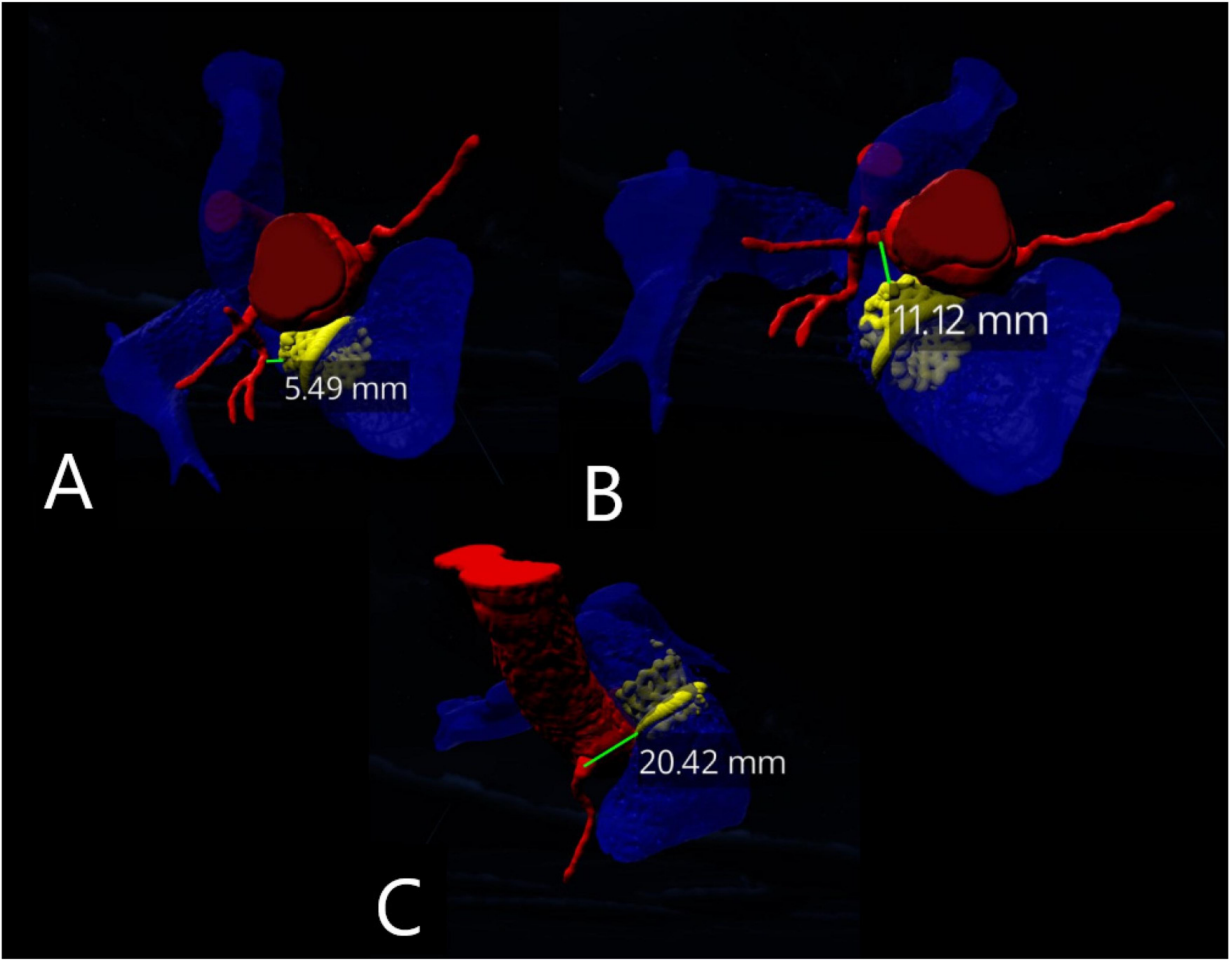
VR-based quantification of conduit–coronary proximity. Representative measurements of the minimal distance between the RV–PA conduit and adjacent coronary arteries obtained within the immersive VR environment. **(A)** Distance to Left Circumflex Artery (LCx), **(B)** Distance to the Left Main Coronary Artery (LMCA), **(C)** Distance to the Right Coronary Artery (RCA).

One interventional cardiologist and one pediatric cardiologist performed VR measurements of the following parameters to assess feasibility and reliability (Figure 3):

1. **Balloon dilation size**: The operators estimated narrowest diameter and maximum stent inflation to determine appropriate balloon size, compared to the actual balloon used in the procedures.
2. **Narrowest diameter**: The cross-sectional diameter of the reconstructed RV-PA conduit measured by both cardiologists.
3. **Conduit inflation**: Virtual RV-PA conduit balloon inflation simulation in 1 mm increments until potential coronary obstruction was identified.
4. **Distance measurements**: Shortest virtual distance between the RV-PA conduit and LCA, LAD, LCX, and RCA as measured by each cardiologist.
5. **Feasibility questionnaire**: Both cardiologists completed a qualitative questionnaire on the value of VR model integration into their procedures. Responses were rated on a scale of 1–5, where 1 = Very unhelpful and 5 = Very helpful.

### Statistical analysis

Several analyses were performed to evaluate measurement accuracy and inter-operator agreement. An intra-class Correlation Coefficient (ICC) was calculated to assess measurement reliability using the Cicchetti and Koo & Li rating scales (22, 23). Pearson correlations and Lin's concordance correlation coefficient (24) compared the operators' measurements of conduit expansions and distances to anatomical landmarks.

The two cardiologists completed questionnaires for each of the 19 cases. Responses were rated on a 5-point scale ranging from 1–Very unhelpful, 2–Unhelpful, 3–Neutral, 4–Helpful, 5–Very helpful. A cutoff of 3 was applied, with ratings ≥4 considered helpful. For the questionnaire analysis, three categories were defined: *High*-both operators rated >3, *Low*-both operators rated ≤3, and *One High*-at least one operator scored >3.

## Results

### Study population and feasibility outcomes

**Figure 4:**
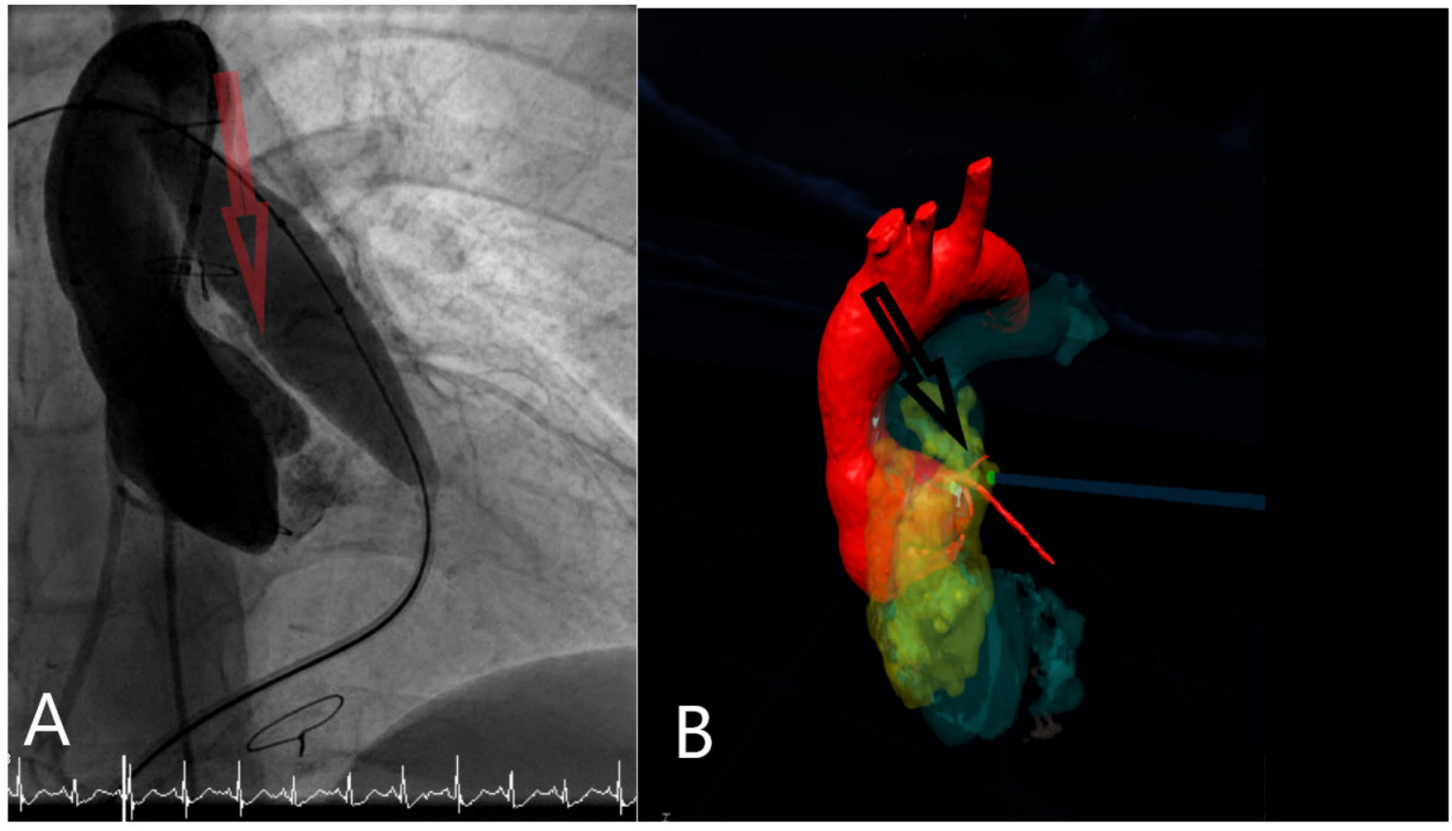
Example case demonstrating coronary compression risk assessment (Red and black arrows respectively). **(A)** Intraprocedural angiographic assessment during test balloon inflation. **(B)** Patient-specific VR model illustrating conduit–coronary proximity and predicted compression risk.

Nineteen patients were enrolled (89.5% male, 10.5% female) with average age 6.4 years (range: 1.2–12.1 years). The study achieved 100% technical feasibility with successful 3D model generation for all patients. Of the cohort, 85% of the patients had been diagnosed with Tetrology of Fallot (TOF) and 15% Transposition of Great Arteries (TGA). Of these, 16 patients (84%) underwent balloon or stent expansion of their conduits. Three patients did not receive stent dilation due to evidence of coronary artery proximity observed during catheterization (Table 1, Figure 4).

**Table 1:** Baseline characteristics of the study population.

Criteria	Values
Age	6.4 ± 3.36 (IQR 3.6–9.2)
Male	17 (89%)
Medical background	TOF (85%), TGA (15%)
Conduit types	14 Homograft (74%), 3 Contegra (15.5%), 2 Hancock (10.5%)
Balloon/stent expansion	16 (84%)

Values are presented as mean ± SD with interquartile range (IQR) for continuous variables and as number (percentage) for categorical variables.

### Inter-operator reliability and measurement accuracy

The retrospective 3D measurements were highly concordant with the actual measurements, thus pointing to the strong feasibility of VR for clinical implementation. The ICC analysis revealed statistically significant agreement between operators for all conduit measurements (Table 2) and distances to LAD and LCX (Table 3). For the conduit size measurements, 66% were rated as excellent on both reliability scales, with the remaining measurements rated as moderate to good. Distance measurements to LAD and LCX demonstrated good to excellent reliability, whereas RCA distance measurements showed moderate reliability.

**Table 2:** Inter-operator agreement for conduit measurements.

Measurement parameter (mm)	Examiner 1 mean	Examiner 2 mean	ICC	95% CI	*P*-value
Narrowest Diameter (*n* = 19)	18.35	18	0.9265	0.666–0.945	<0.001
Conduit Expanded (*n* = 19)	3	3	0.743	0.173–0.819	0.009
Estimated Balloon Diameter (*n* = 19)	N/A	N/A	0.915	0.632–0.938	<0.001

**Table 3:** Inter-operator agreement for coronary distance measurements.

Measurement parameter (mm)	Examiner 1 mean	Examiner 2 mean	ICC	95% CI	*P*-value
Distance to LCA (*n* = 19)	27	9.04	0.277	−0.244–0.936	0.135
Distance to LAD (*n* = 19)	9.15	8.5	0.829	0.432–0.894	<0.001
Distance to LCx (*n* = 19)	18.4	14.5	0.771	0.234–0.839	0.004
Distance to RCA (*n* = 19)	19.3	13	0.550	−0.098–0.707	0.116

### User experience and clinical utility assessment

Both cardiologists reported a consistently positive experience using the VR models, with no questionnaire item receiving a low score (≤3). The overall mean usability score was 3.97 ± 0.52 for operator 1 and 4.10 ± 0.24 for operator 2, yielding a combined mean of 4.04 ± 0.41 across all evaluations.

Inter-operator agreement across the nine usability questions was generally strong. Seventy-eight percent of the items were at least moderately correlated between operators, and 28.5% showed high correlations. Pearson correlation coefficients across the questionnaire ranged from 0.26 to 0.74, indicating acceptable consistency in operator scoring. When using a threshold of >3 to define a “high” rating, 88% of the questions were rated as high by at least one operator, and 62% were rated as high by both. Score dispersion was low across items, with standard deviations ranging from 0.24 to 0.52, reflecting stable operator perceptions of VR usability.

The strongest concordance was observed on items evaluating whether VR would influence procedural planning; specifically, whether the cardiologist would proceed with conduit dilation based on the VR visualization and whether they would prefer incorporating VR or CT/VR models into future procedures. The lowest agreement was observed for the item assessing the usefulness of virtual conduit dilation; however, both operators still rated this feature positively.

### Comparison of CT vs. VR utility

When comparing CT to VR (Table 4), the cardiologists rated VR significantly higher for assessing the coronary artery course (4.575 vs. 3.775, *p* < 0.0001). However, VR did not significantly outperform CT in influencing the decision to proceed with dilation (3.55 vs. 3.90, *p* = 0.169).

**Table 4:** Comparison of CT vs. VR mean scores on the qualitative questionnaire comparing cardiologist ratings of the utility of CT vs. VR on 2 key questions exploring the course of coronary artery and the impact on the dilation decision.

CT vs. VR comparison	CT mean score	VR mean score	*P*-value
Course of coronary artery	3.775	4.575	*<0.0001*
Dilation decision	3.9	3.55	0.169

## Discussion

This bi-center feasibility study suggests that patient-specific 3D VR modeling can be effectively integrated into the pre-procedural planning of RV-PA conduit stenting, and demonstrates advantages over conventional CT imaging. VR enabled reliable reconstruction of complex cardiac anatomy from routine CT datasets, with high inter-operator agreement across most measurement parameters (12–30). These findings suggest that VR provides a technically reproducible approach for evaluating conduit–coronary spatial relationships. Importantly, both cardiologists consistently rated VR as more helpful than CT alone, particularly for visualizing coronary artery trajectories (31, 32).

While promising, this study is limited by its small sample size, retrospective design, and the absence of real-time validation during catheterization. Additionally, our VR simulations are based on static CT imaging and are therefore rigid anatomy-based models and do not incorporate flow (e.g., computational fluid dynamics models) or tissue mechanical properties, which may affect absolute accuracy. Despite these limitations, the results point to VR as a feasible, intuitive, and clinically meaningful adjunct to standard imaging.

### Technical feasibility and clinical implementation

This study achieved 100% technical success in generating patient-specific 3D models from routine clinical CT datasets, thus confirming that VR can be reliably integrated into existing imaging workflows. The strong inter-operator agreement (ICC >0.7 across most measurements) further supports the reliability required for clinical adoption, consistent with recent evidence demonstrating VR's capacity to improve accuracy and reduce procedure time in cardiac catheterization (20).

The high user satisfaction (mean 4.04/5) and uniformly positive qualitative feedback indicated that VR systems are readily usable by experienced cardiologists. This is notable given the broader challenges that still limit clinical VR deployment, including multimodality imaging integration and the need for real-time procedural visualization (17). Overall, these findings suggest that VR is both technically feasible and well-positioned for wider implementation in congenital interventional planning.

### VR advantages over conventional imaging

The findings underscore the advantages of VR over conventional CT imaging, most notably in visualizing coronary artery pathways (mean 4.58 vs. 3.78, *p* < 0.0001). This finding is consistent with evidence that VR and 3D visualization enhance depth perception and spatial understanding, both of which are critical elements in planning complex cardiac interventions (25). By enabling immersive inspection of patient-specific anatomy, VR addresses a key limitation of CT, which requires the operator to mentally reconstruct 3D relationships from 2D images.

The improved spatial awareness afforded by VR translated into tangible clinical benefits in our cohort, particularly for assessing coronary course and, to a lesser extent, coronary–conduit proximity. These results suggest that the theoretical advantages of extended reality technologies may yield meaningful improvements in anatomic clarity and pre-procedural planning.

Overall, VR appeared to add value for spatial understanding of coronary anatomy; however, final decisions regarding conduit dilation remained multifactorial and dependent on the integrated clinical and imaging context.

### Clinical impact and risk mitigation

The principal clinical value of VR in RV-PA conduit planning lies in its ability to enhance pre-procedural risk assessment, especially for identifying patients who could be at higher risk of coronary artery compression. By providing patient-specific visualization of coronary arteries and conduit–coronary spatial relationships, VR can simplify complex anatomic proximity more so than with conventional 2D CT imaging. In this way, VR could support more detailed risk stratification, inform patient selection, and contribute to safer procedural decision-making (33).

VR may also have practical workflow benefits in the catheterization laboratory. Pre-identifying optimal viewing angles and mentally rehearsing a procedural roadmap could reduce iterative angiographic runs, with the potential to decrease fluoroscopy time and radiation exposure while improving efficiency. Consistent with this, operators in our study rated VR as highly valuable for procedural planning; however, overall decision-making ratings were similar to CT, suggesting VR functioned primarily as a complementary qualitative tool rather than a replacement for standard CT-based assessment and final intraprocedural clinical evaluation. While our findings indicate that VR provides meaningful added value for spatial understanding of coronary anatomy, final decisions regarding conduit dilation remained multifactorial and dependent on the integrated clinical and imaging context.

### Broader implications for cardiovascular care

This feasibility study contributes to the growing body of evidence supporting the role of immersive technologies in cardiovascular medicine. Augmented and virtual reality tools are increasingly being recognized for their value in diagnosis, medical training, and procedural planning by providing detailed, intuitive, and fully interactive visualization of cardiac structures (26). Our successful use in a pediatric cohort underscores VR's versatility and suggests it could be applied across a broad spectrum of ages, anatomical variants, and procedural complexity particularly as a pre-intervention simulation tool for procedural rehearsal.

Beyond anatomical assessment, VR offers clinicians a unique “flight simulator” environment in which interventionalists can rehearse procedural strategies, explore multiple approaches, and anticipate anatomical challenges without exposing patients to risk. Our findings provide early support for this concept in the context of RV-PA conduit interventions, suggesting that VR may ultimately serve as a powerful adjunct to enhance operator preparedness, refine procedural planning, and improve patient safety.

### Evolving RV-PA conduit interventions

Historically, RV-PA conduit interventions relied on angiography-guided balloon dilation and early stenting to prolong conduit lifespan, in the absence of cross-sectional anatomical assessment (34–38). Surgical durability studies further characterized conduit degeneration and reintervention patterns but offered limited guidance on coronary risk stratification (12, 27–30). More recent pediatric RVOT stenting series have reported favorable outcomes, though primarily in native RVOT obstruction rather than valved conduits. Early conduit stenting studies predated modern CT-based approaches and thus could not anticipate conduit-coronary interactions, despite the well-acknowledged risk of coronary compression during dilation (12, 13, 31, 32). Advances in CT-derived 3D reconstruction and immersive visualization have considerably enhanced preprocedural planning in congenital and structural heart interventions (14–26). However, to the best of our knowledge, no RV–PA conduit study has incorporated patient-specific VR reconstructions or simulated dilation.

By integrating VR with conventional CT, our study proposes a practical workflow framework that may enhance assessment of the coronary artery course and conduit–coronary proximity, thereby supporting more informed pre-procedural planning.

### Study limitations and future directions

Several limitations should be acknowledged. First, the small sample size (*n* = 19) limits generalizability and reduces statistical power to detect more subtle effects. Second, there is no universally accepted gold standard for quantifying spatial relationships in RV-PA conduit anatomy, making external validation inherently challenging and highlighting the broader need for standardized protocols and guidelines for VR use in clinical research. Third, this study assessed static anatomical models and did not incorporate cardiac motion patient-specific tissue/mechanical properties of the conduit and coronary arteries. Likewise, we did not perform dynamic assessments (e.g., computational modeling such as CFD or formal physiologic testing), reflecting the study's focus on anatomic relationships rather than functional effects and underscoring the ongoing need for intraprocedural dynamic evaluation. Future work should integrate dynamic imaging and modeling approaches and assess whether VR-guided planning translates into measurable improvements in procedural outcomes and safety. Fourth, we did not evaluate cost-effectiveness, which remains an important consideration for broader clinical adoption.

The moderate inter-operator reliability observed for RCA distance measurements suggests that further refinement of the measurement protocols or anatomical reference points may be warranted. Additionally, although user satisfaction was high, longer-term evaluation of learning curves, operator performance, and sustained utilization patterns are crucial to guiding effective implementation.

## Conclusion

This bi-center feasibility study provides compelling evidence that patient-specific 3D virtual reality (VR) modeling is a technically reliable and clinically meaningful adjunct for pre-procedural planning in RV-PA conduit interventions. VR successfully generated anatomic models from routine CT data in all cases and demonstrated strong inter-operator reliability across most parameters, supporting its feasibility for standardized implementation. Compared with conventional CT review, VR was perceived to offer added value most notably for coronary artery assessment where operators rated VR significantly superior for visualizing coronary trajectories and course.

Beyond static imaging, VR's immersive 3D environment enabled the dynamic simulation of balloon expansion, thus facilitating clearer identification of high-risk anatomical configurations prior to catheterization. These capabilities point to the meaningful potential of VR in reducing procedural complications, decreasing radiation exposure through improved pre-defined imaging angles, and supporting safer, more informed decision-making in complex congenital cardiac interventions. The high user satisfaction expressed by the experienced cardiologists further underscores the practical readiness of VR systems for clinical adoption.

Despite these promising results, broader validation is required. Larger prospective studies are needed to determine the predictive accuracy of VR-based assessments, quantify its impact on procedural outcomes, and establish evidence-based guidelines for integration into clinical workflows. Additional developments, including incorporation of dynamic testing, multimodality imaging, and cost-effectiveness analyses, will be essential as the technology matures.

VR represents a powerful and evolving tool with the potential to enhance anatomical understanding, improve procedural planning, and mitigate risk in pediatric RV-PA conduit interventions. As immersive technologies continue to advance, VR is poised to become an integral imaging component, while it is clear that current protocols serve as strong adjuncts and not a replacement for the final clinical assessment and decision-making which takes place during the invasive procedure.

## Data Availability

The original contributions presented in the study are included in the article/Supplementary Material, further inquiries can be directed to the corresponding author.

## Appendix 1. Feasibility assessment questionnaire.

Please respond to the questions below on a scale from 1 to 5 where 1 = Very unhelpful, to 5 = Very helpful.

1. How well were you able to estimate the path of the coronary arteries based on the CT scan?
2. How well were you able to estimate the path of the coronary arteries based on the VR model?
3. How helpful was it to dilate the conduit on the CT?
4. How helpful was it to dilate the conduit on the VR model?
5. How much would the VR model contribute to your decision to catheterize the patient?
6. In the future, how helpful would it be to see the VR model prior to the procedure?
7. How helpful would it be to have the VR model available during the procedure?
8. How helpful was the VR model in predicting complications during the procedure?
9. Overall, how helpful was the VR model compared to the standard CT?

## Appendix 2. Mean scores per Question

**Table:**

Question	Mean Score
How well were you able to estimate the path of the coronary arteries based on the CT scan?	3.7750
How well were you able to estimate the path of the coronary arteries based on the VR model?	4.5750
How helpful was it to dilate the conduit on the CT?	3.9000
How helpful was it to dilate the conduit on the VR model?	3.5500
How much would the VR model contribute to your decision to catheterize the patient?	4.1750
In the future, how helpful would it be to see the VR model prior to the procedure?	4.5000
How helpful would it be to have the VR model available during the procedure?	4.1000
How helpful was the VR model in predicting complications during the procedure?	3.5250
Overall, how helpful was the VR model compared to the standard CT?	4.2500
